# Karyopherin α7 (KPNA7), a divergent member of the importin α family of nuclear import receptors

**DOI:** 10.1186/1471-2121-11-63

**Published:** 2010-08-11

**Authors:** Joshua B Kelley, Ashley M Talley, Adam Spencer, Daniel Gioeli, Bryce M Paschal

**Affiliations:** 1 Center for Cell Signaling, University of Virginia, Charlottesville, VA, 22908, USA; 2 Department of Microbiology, University of Virginia, Charlottesville, VA, 22908, USA; 3 Department of Biochemistry and Molecular Genetics, University of Virginia, Charlottesville, VA, 22908, USA

## Abstract

**Background:**

Classical nuclear localization signal (NLS) dependent nuclear import is carried out by a heterodimer of importin α and importin β. NLS cargo is recognized by importin α, which is bound by importin β. Importin β mediates translocation of the complex through the central channel of the nuclear pore, and upon reaching the nucleus, RanGTP binding to importin β triggers disassembly of the complex. To date, six importin α family members, encoded by separate genes, have been described in humans.

**Results:**

We sequenced and characterized a seventh member of the importin α family of transport factors, karyopherin α 7 (KPNA7), which is most closely related to KPNA2. The domain of KPNA7 that binds Importin β (IBB) is divergent, and shows stronger binding to importin β than the IBB domains from of other importin α family members. With regard to NLS recognition, KPNA7 binds to the retinoblastoma (RB) NLS to a similar degree as KPNA2, but it fails to bind the SV40-NLS and the human nucleoplasmin (NPM) NLS. KPNA7 shows a predominantly nuclear distribution under steady state conditions, which contrasts with KPNA2 which is primarily cytoplasmic.

**Conclusion:**

KPNA7 is a novel importin α family member in humans that belongs to the importin α2 subfamily. KPNA7 shows different subcellular localization and NLS binding characteristics compared to other members of the importin α family. These properties suggest that KPNA7 could be specialized for interactions with select NLS-containing proteins, potentially impacting developmental regulation.

## Background

Eukaryotic cells are defined by the separation of DNA from the rest of the cell by the nuclear envelope, a double bilayer made selectively permeable by Nuclear Pore Complexes (NPC) [1]. Transport of proteins between the nucleus and the cytoplasm is carried out by karyopherins, a family of proteins made up of importins and exportins [2,3]. Classical nuclear localization signal (NLS) dependent nuclear import is carried out by importin α and importin β Importin α family members bind NLS cargo, and bind to importin β through an N-terminal importin β binding domain (IBB). Importin β mediates translocation of the NLS-Importin α-Importin β import complex into the nucleus through direct interactions with the NPC. Once in the nucleus, RanGTP binds to importin β and induces dissociation of the import complex [4]. Exportin mediated nuclear export is regulated by RanGTP through a related mechanism. Whereas RanGTP dissociates import complexes by binding importins, exportins must bind to RanGTP in order to bind nuclear export signal (NES) containing cargoes [5]. The heterotrimeric export complex then translocates through the NPC and is dissociated in the cytoplasm by RanGAP stimulated conversion of RanGTP to RanGDP.

While there are at least 10 importin β family members which can bind directly to cargo and mediate import [4], importin β is unique in its ability to bind the importin α family of nuclear transport receptors (also called karyopherin α) [2,3]. Importin α binds to two major classes of NLS, both characterized by basic amino acids; a monopartite NLS, such as the SV40 NLS, which consists of a single cluster of basic amino acids; and a bipartite NLS, such as the retinoblastoma (RB) NLS, which consists of two clusters of basic amino acids, separated by a ~10 residue spacer [6]. The architecture of importin α proteins is composed of Armadillo (ARM) repeats, a three a-helix motif named for the *D. melanogaster *homologue of β catenin [7]. The binding site for a monopartite NLS is located between the 2^nd ^and 4^th ^ARM repeats and is called the major site [8]. Importin α binds to the C-terminus of bipartite NLS sequences with the major site and to the N-terminal element of the bipartite NLS using a smaller site created by the 7^th ^and 8^th ^ARM repeats called the minor site [8-10]. The accessibility of these NLS binding sites is regulated by an autoinhibitory mechanism. The IBB of importin α contains basic amino acids that bind to the NLS binding surface when the receptor is in an autoinhibited state [11-14]. Importin α binding to NLS cargo and to importin β is, therefore, a cooperative process because importin β binding to the IBB relieves the autoinhibition of importin α. Relief of autoinhibition facilitates Importin α binding to NLS cargo. After nuclear import, the complex is dissociated by the cooperative effects of RanGTP binding to importin β, and binding of importin α to CAS [15]. CAS is an exportin which forms a trimeric complex consisting of CAS, RanGTP and importin α, and is responsible for recycling importin α to the cytoplasm [16].

Yeasts encode a single importin α, but higher eukaryotes encode three importin α subfamilies, designated importin α1, α2, and α3. There are six previously described human importin α forms, each encoded by different gene. Importin α family members show preferences for specific types of NLS cargo [17-20], although there is also some functional redundancy. Most NLSs are very similar in sequence leading to the suggestion that importin α specificity is conferred by the protein context of the NLS imparted by the cargo [21]. It appears that additional importin α proteins are required for a multicellular organism because different importin α's have specific cargoes to import during differentiation [22]. Studies in *D. melanogaster *have shown that importin α2 is not required in adult flies, but is required for oogenesis [23] and that importin α3 is favored for a step in larval development [24]. Similarly, it has been shown in *C. elegans *that while importin α3 is ubiquitously expressed, α1 and α2 are expressed only in the germline, and are not sufficient to replace α3 in somatic cells [25]. In mouse embryonic stem cells, switching from importin α1 (KPNA2) expression to importin α5 (KPNA1) expression has been shown to induce neural differentiation [26]. These data suggest that cargo interactions with specific importin α isotypes are important for controlling differentiation pathways.

Here we describe a novel importin α in humans, the seventh member of the importin α family, which we term karyopherin alpha 7 (KPNA7). This new α family member is most similar to KPNA2 and a member of the α2 subfamily. We show that KPNA7 binds to importin β through an N-terminal IBB, but that its importin β binding affinity appears to be much higher than that of the other KPNAs. KPNA7 localization is predominantly nuclear in HeLa cells, highlighting another difference between 7 and the other importin α family members.

## Results

### KPNA7 is a novel member of the Importin Alpha family

**Figure 1:**
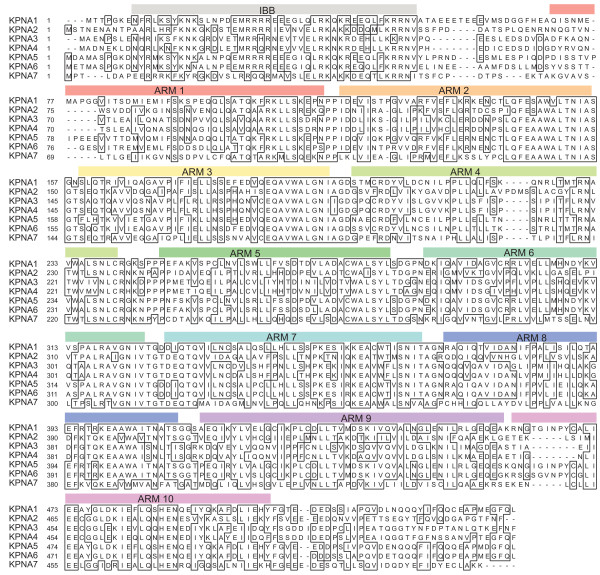
**ClustalW alignment of the seven KPNA proteins that comprise the importin α family in humans**. Positions of the armadillo repeats (ARM 1-10) and the importin β binding (IBB) domain are indicated with colored boxes. Amino acids which are identical in ≥ 4 of the KPNA proteins are boxed.

There are six known members of the human importin α family. We used BLAST to query the human genome with the importin α family member KPNA2, and retrieved a sequence predicted to encode a seventh member of the importin α family. Because the sequence of the putative importin α was generated by gene prediction, we set out to experimentally validate the expression and function of the predicted importin α gene [GenBank:XM_376655] as an importin α. Using RT-PCR, we amplified a product of the expected size from RNA prepared from the prostate cancer cell line LNCaP. We sequenced the cDNA [Genbank:EU126604], and found that it matched that of the predicted gene, leading us to conclude that the gene is transcribed in human cells. This gene is the seventh member of the importin α family of transport receptors, therefore we named it karyopherin alpha 7 (KPNA7). The complete human importin α family (KPNA1-7) is shown in Table 1 with their previous designations, as well as accession and Gene ID numbers. Alignment of the KPNA7 protein sequence with KPNA's 1-6 using ClustalW (Fig 1) reveals that KPNA7 is 54.7% identical to its closest importin α family member, the prototypical KPNA2 (Rch1). The maximum identity between KPNA7 and members of the α1 and α3 subfamilies is only 43.7% (Table 2). KPNA1, KPNA5, and KPNA6 share a minimum of 80.7% identity, while KPNA3 and KPNA4 are 85.8% identical, emphasizing that KPNA7 is highly divergent from other importin α family members. The multiple alignment (Fig. 1) shows, however, that KPNA7 is related to other importin α family members throughout the length of the protein, suggesting that it is an NLS binding protein.

**Table 1:** Importin α family identity matrix.

	KPNA 1	KPNA 2	KPNA 3	KPNA 4	KPNA 5	KPNA 6	KPNA 7
**KPNA 1**	**100**	45.7	47.9	47.5	80.7	82.1	42.6

**KPNA 2**		**100**	49.5	50.9	48.1	47.3	54.7

**KPNA 3**			**100**	85.8	48.4	47.8	43.7

**KPNA 4**				**100**	48.7	48.2	43.7

**KPNA 5**					**100**	84.5	41.4

**KPNA 6**						**100**	42.5

**KPNA 7**							**100**

The number shown is the percent identity between the respective KPNA proteins.

**Table 2:** KPNA and importin α nomenclature.

KPNA	Importin α	Accession	GeneID	Chr Locus
1	5	NM_002264.3	3836	3q21

2	1	NM_002266.2	3838	17q24

3	4	NM_002267.3	3839	13q14

4	3	NM_002268.3	3840	3q25

5	6	NM_002269.2	3841	6q22

6	7	NM_012316.4	23633	1p35

7	8	NM_001145715.1	402569	7q22

Here we show the KPNA numbering system and the corresponding importin alpha numbers, as well as unique identifiers: Accession Number, Gene ID number, and the chromosomal locus.

**Figure 2:**
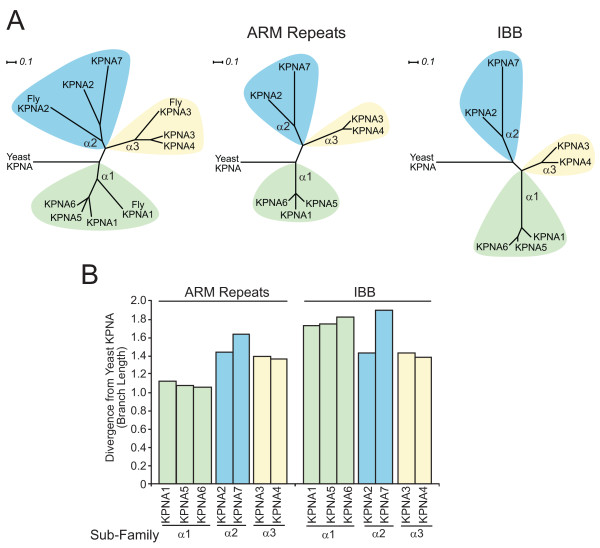
**Phylogenetic analysis of the importin α family**. (A) Phylogenetic trees generated using the KPNA protein sequences from human, D. melanogaster (fly), and S. cerevisiae (Yeast). The three subfamilies are shaded in green (a1 subfamily), blue (a2 subfamily) and yellow (a3 subfamily). Phylogenetic trees were also generated using the ARM repeat domains and the IBB domains. Scales bar is 0.1 branch length. of the human importin a family. The sole S. cerevisiae importin α, SRP1 (Yeast KPNA), is included as a common ancestor. D. melanogaster contains one member each of the α1, α2, and α3 subfamilies, which are included (Fly KPNA) to define the subfamilies. The phylogenetic trees labeled ARM repeats and IBB were generated using either the ARM repeat or IBB domains of all 7 human KPNA proteins. (B) Comparison of the divergence of the IBB and ARM repeat domains of the human KPNA proteins from the same domains in Yeast SRP1. Divergence was measured as branch length of the phylogenetic trees from SRP1 to the given domain of each KPNA.

KPNA7 homologues are found in vertebrates including mouse (62.1% identity, NP_001013796.2), xenopus (59.6% identity, NP_001081744.1) and zebra fish (51.9% identity, NP_998235.1). In all animals, importin α proteins are classified into 3 subfamilies, α1, α2, and α3 [23,27]. While vertebrates have seven importin α family members, invertebrates such as Drosophila have only 3 importin α proteins, one for each subfamily. We created a phylogenetic tree of the complete human importin α family using Protdist (Fig. 2A). The *Drosophila melanogaster *importin α proteins were included in the phylogram (Fig 2A) to help define each subfamily. We included the sole *S. cerevisiae *importin α, SRP1, in the tree to illustrate the relationships of the human KPNA proteins to a common ancestor. The phylogenetic tree shows that KPNA7 is a member of the α2 subfamily.

The alignment of KPNA7 with KPNA's 1-6 (Fig 1) revealed that the IBB of KPNA7 has a region of low identity to the consensus. Separate phylogenetic analysis of the ARM repeats and IBB of the KPNAs show that the KPNA7 IBB is more divergent than the body of KPNA7 (Fig. 2B,C), as the branch length separating the KPNA7 IBB from S. cerevisiae importin α is longer than the branch length to the KPNA7 body. The α1 subfamily has the least divergent body, while the IBBs of this subfamily are almost as divergent as the KPNA7 IBB. KPNA2, KPNA3, and KPNA4 show the same branch lengths for both their bodies and IBB's, suggesting they evolved at the same rate. KPNA7 has an N-terminal domain which is 48% identical to the KPNA2 IBB, and only 24% identical to the KPNA5 IBB. The strong difference in the IBB of KPNA7 from all other KPNAs suggests that the KPNA7 IBB is has diverged in response to different selective pressures.

### The N-terminus of KPNA7 is a functional IBB

**Figure 3:**
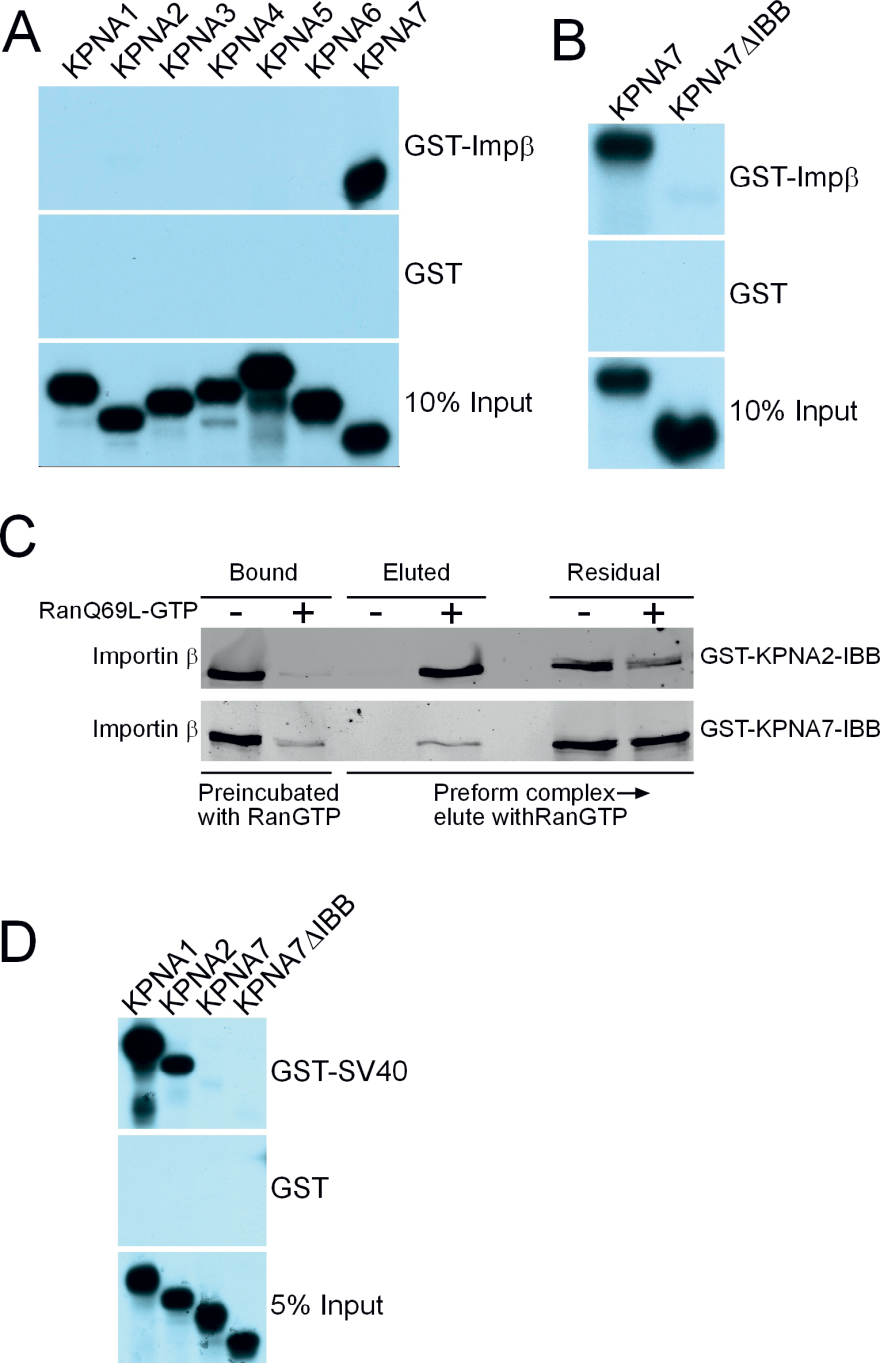
**KPNA7 contains an N-terminal IBB**. (A) KPNA proteins were translated as ^35^S-methionine labeled polypeptides and used in binding assays with GST-Importin β. (B) Full-length and ΔIBB forms of KPNA7 tested for binding to Importin β. (C) Importin β binding to the IBB of KPNA7 is RanGTP-sensitive. Recombinant Importin β binding to GST-IBB proteins was performed in the absence and presence of recombinant RanGTP, and by subsequent addition of RanGTP to preformed GST-IBB-importin β complexes. These assays were performed with the GTPase deficient RanQ69L mutant. Bound and eluted fractions were examined by immunoblotting using a monoclonal antibody to Importin β. (D) KPNA1, KPNA2, KPNA7, KPNA7 ΔIBB binding to GST-SV40 NLS using ^35^S-methionine labeled polypeptides.

Importin α proteins act as adaptors for nuclear import by binding both NLS cargo and importin β. Importin α binding to importin β is mediated by the N terminal IBB domain, making possible the α/β heterodimer capable of carrying NLS cargo into the nucleus. The low sequence identity of the KPNA7 IBB with other KPNA IBB domains could be indicative of changes in function, and so we tested whether the N-terminus of KPNA7 acts as an IBB. We examined KPNA7 binding to importin β by immobilizing GST-importin β on glutathione beads, and incubating the beads with *in vitro *translated, ^35^S-Methionine labeled KPNA proteins (Fig 3A). Surprisingly, KPNA7 displayed the most stable binding to the GST-Importin β. A longer exposure of the film reveals a low level of KPNA1-6 binding to importin β. Because the autoinhibited state and importin β binding are mutually exclusive, the low level of KPNA1-6 binding to importin β likely reflects the stability of the autoinhibited state of these proteins. In order to confirm that the β binding of KPNA7 is conferred by its IBB region, we performed a GST pulldown assay using in vitro translated, ^35^S-Methionine labeled proteins; KPNA7, and a mutant KPNA7 in which the putative IBB had been removed (KPNA7ΔIBB). Deletion of the KPNA7 IBB resulted in complete loss of KPNA7 binding to importin β (Fig 3B).

Because reticulocyte lystate contains proteins (including transport factors) that might influence the outcome of these binding assays, we used recombinant proteins to formally test whether the N-terminus of KPNA7 functions as an IBB. GST fusions of the IBB domains from KPNA2 and KPNA7 were immobilized on glutathione beads, recombinant importin β was added, and bound fractions analyzed by immunoblotting. Importin β bound to the IBB domains of both KPNA2 and KPNA7; the interactions were Ran-sensitive because they were reduced significantly by including RanQ69L (preloaded with GTP) in the binding reaction (Fig. 3C, first two lanes). We also found that preformed complexes of importin β with IBB domains of KPNA2 and KPNA7 were dissociated by addition of RanGTP (Fig. 3C), a reaction that mimics Ran-dependent disassembly of import complexes. Based on semi-quantitative immunoblotting, RanGTP addition resulted in dissociation of 60% of the preformed importin β-KPNA2 complexes but only 3% of the preformed importin β- KPNA7 complexes. This result, together with data showing that full-length KPNA7 binds efficiently to importin β (Fig. 2A), suggests that KPNA7 import complexes could be more stable than complexes formed between importin β and other KPNA proteins.

The competitive autoinhibition of NLS binding by the IBB may be altered by changes in the IBB affinity for importin α. Thus we decided to test whether the KPNA7ΔIBB would show enhanced binding to the SV40-NLS compared to full length KPNA7. Unexpectedly, neither full length nor IBB deletion forms of KPNA7 display significant levels of binding to the SV40 NLS (Fig 3D). The difference in SV40 NLS binding affinities between KPNA1 or KPNA 2 and KPNA7 suggests that there may be a difference in their NLS binding surfaces.

**Figure 4:**
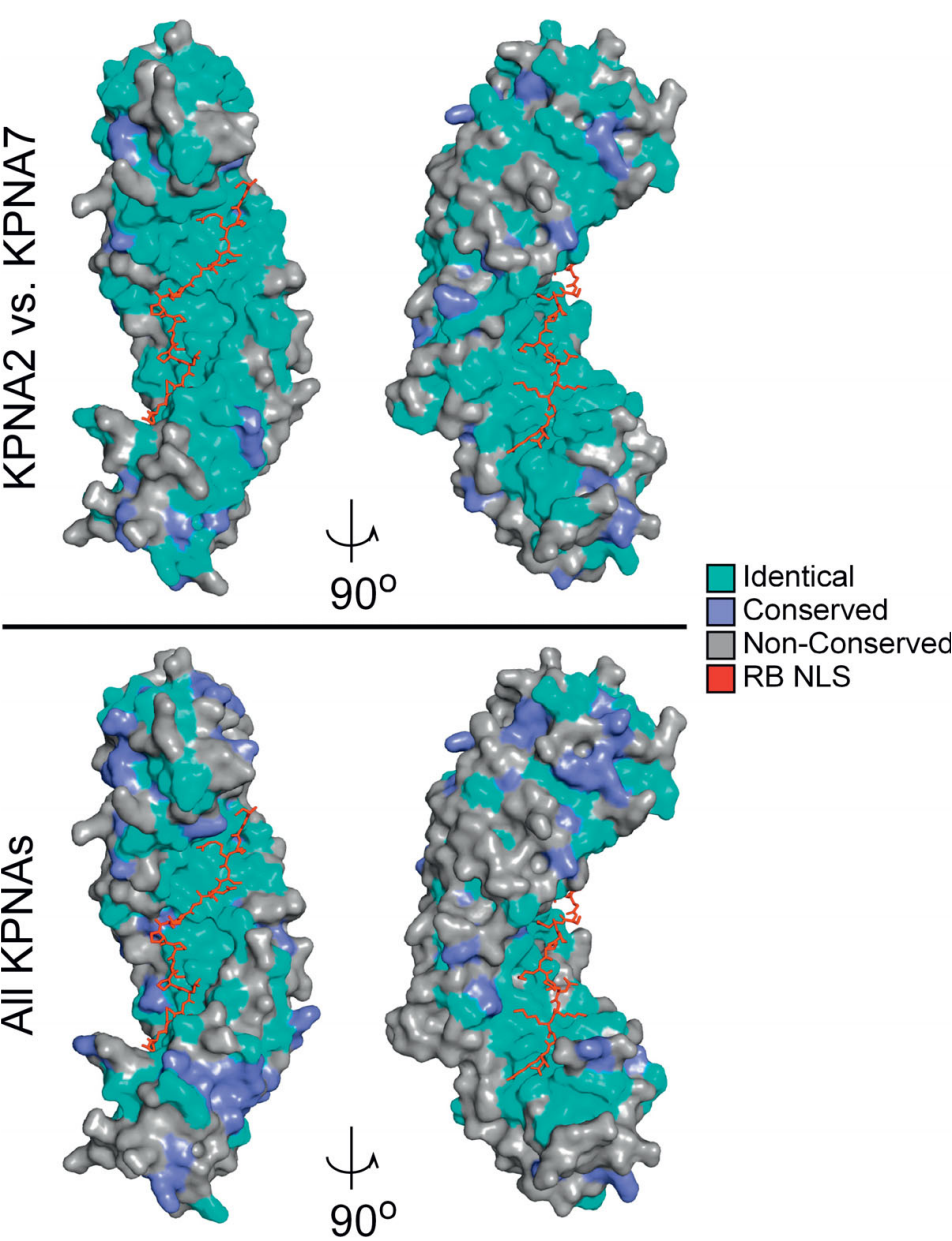
**Structural model illustrating the conservation of amino acids that create the NLS binding surface in KPNA proteins**. The ClustalW alignment of KPNA2 and KPNA7 (Top panel) or all of the KPNA's (bottom panel) was used to map identical (cyan), conserved (blue), and different (gray) residues onto the importin α/RB NLS (Red) structure 1PJM [30]. The left image is rotated 90° and shown on the right. Images were created using Pymol http://www.pymol.org/.

Crystal structures have been solved for several importin α proteins, yeast SRP1 [8], mouse and human KPNA2 [13,28], and human KPNA1 [29]. The network of amino acids that create the NLS binding surface have been described in detail for the SV40 NLS, RB NLS, and nucleoplasmin (NPM) NLS, among others [8-10,30,31]. We used molecular modeling to examine the conservation of the NLS binding surface between KPNA2 and KPNA7. We mapped identical and conserved residues between KPNA2 and KPNA7 (Fig. 4A) as well as KPNAs 1 through 7 (Fig. 4B) onto the structure of mouse KPNA2 co-crystalized with the bipartite RB NLS (PDB ID: 1PJM , [30]). The RB NLS binds both the major and minor binding sites on importin α, and thus serves as a reference for the entire NLS binding surface [9]. The residues in KPNA2 that bind the RB NLS are identical in KPNA7. Moreover, the NLS binding surface is almost perfectly conserved in all seven KPNA proteins. 22/22 amino acids that interact with the RB NLS [30] and 16/17 which interact with the SV40 NLS [31] are perfectly conserved among the human KPNA proteins (Fig. 4B). The only amino acid involved in SV40 NLS binding that is not identical in all family members is R106 in KPNA2, which corresponds to K112 (KPNA1); S101 (KPNA3); S101 (KPNA4); K113 (KPNA5); K110 (KPNA6); and Q100 (KPNA7). Notably, conservation at this position is observed within the α1 and α3 subfamilies.

### KPNA 7 shows limited RB NLS binding and fails to bind to either the SV40 NLS or the nucleoplasmin (NPM) NLS

**Figure 5:**
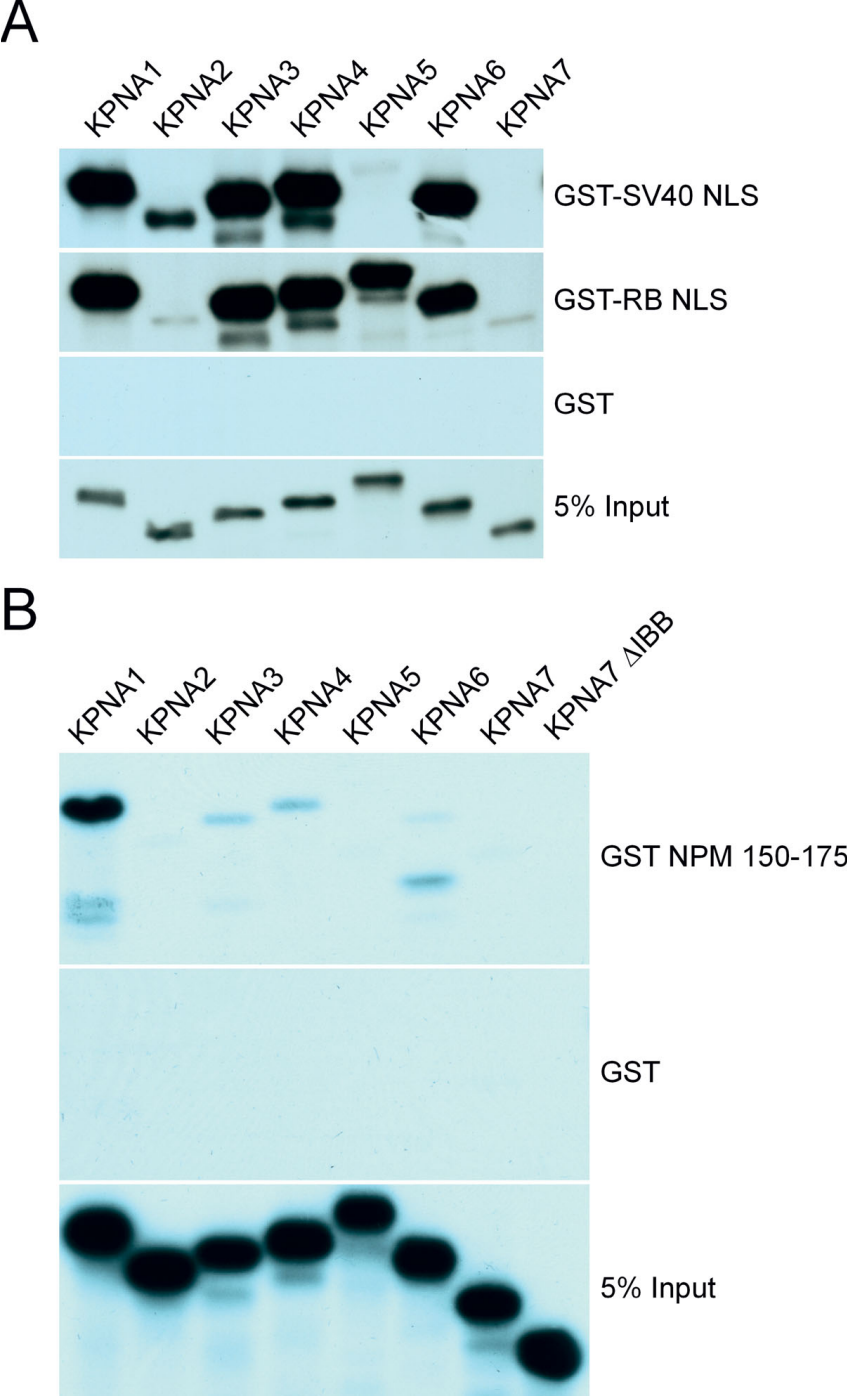
**Monopartite and bipartite NLS binding of the importin α family**. Binding reactions between in vitro translated 35 S methionine labeled human KPNA's in solution and (A) GST-SV40 NLS (monopartite) and GST-RB NLS (bipartite) immobilized upon glutathione agarose beads, or (B) GST-NPM NLS (NPM 150-175). As a negative control, GST was used alone.

KPNA1, KPNA2, KPNA3, KPNA4, and KPNA6 import cargo containing the SV40 NLS in permeabilized cell assays [19]. The perfect conservation of amino acids that form the RB NLS binding surface on KPNAs 1 through 7 suggests these receptors should have similar affinities for RB NLS. To examine the RB NLS and SV40 NLS binding characteristics of the entire importin α family, we performed binding assays with in vitro translated ^35^S Methionine-labeled KPNA proteins (Fig. 5A). The target NLS proteins were expressed as GST fusions and bound to glutathione beads. KPNA2 and KPNA7 (α2 subfamily) bound to the RB NLS weakly, while KPNA1, KPNA5, and KPNA6 (α1 subfamily) bound the RB NLS at a high level. The SV40 binding was more complex, in that it did not track with subfamily, as KPNA1, KPNA3, KPNA4, and KPNA6 bound the SV40 NLS strongly; KPNA2 bound less well; and KPNA5 and KPNA7 failed to bind the SV40 NLS altogether. Notably, RB NLS binding appears to be consistent within subfamilies, while the SV40 NLS binding showed binding differences within subfamilies.

After we deposited the human KPNA7 sequence to GenBank (EU126604), Tejomurtula et al. reported the sequence of bovine KPNA7 and showed that it binds bovine nucleoplasmin [32]. We therefore tested the ability of human KPNA7 to bind the human NPM NLS (NPM 150-175) (Fig 5B). The NPM NLS bound to KPNA1 very strongly, and showed some binding to KPNA3, KPNA4, and KPNA6 (although most of the bound KPNA6 appears to be a degradation product). Under our reaction conditions, neither KPNA7 nor KNPA7ΔIBB bound to the human NPM NLS.

### KPNA7 Localizes to the Nucleus in HeLa cells

**Figure 6:**
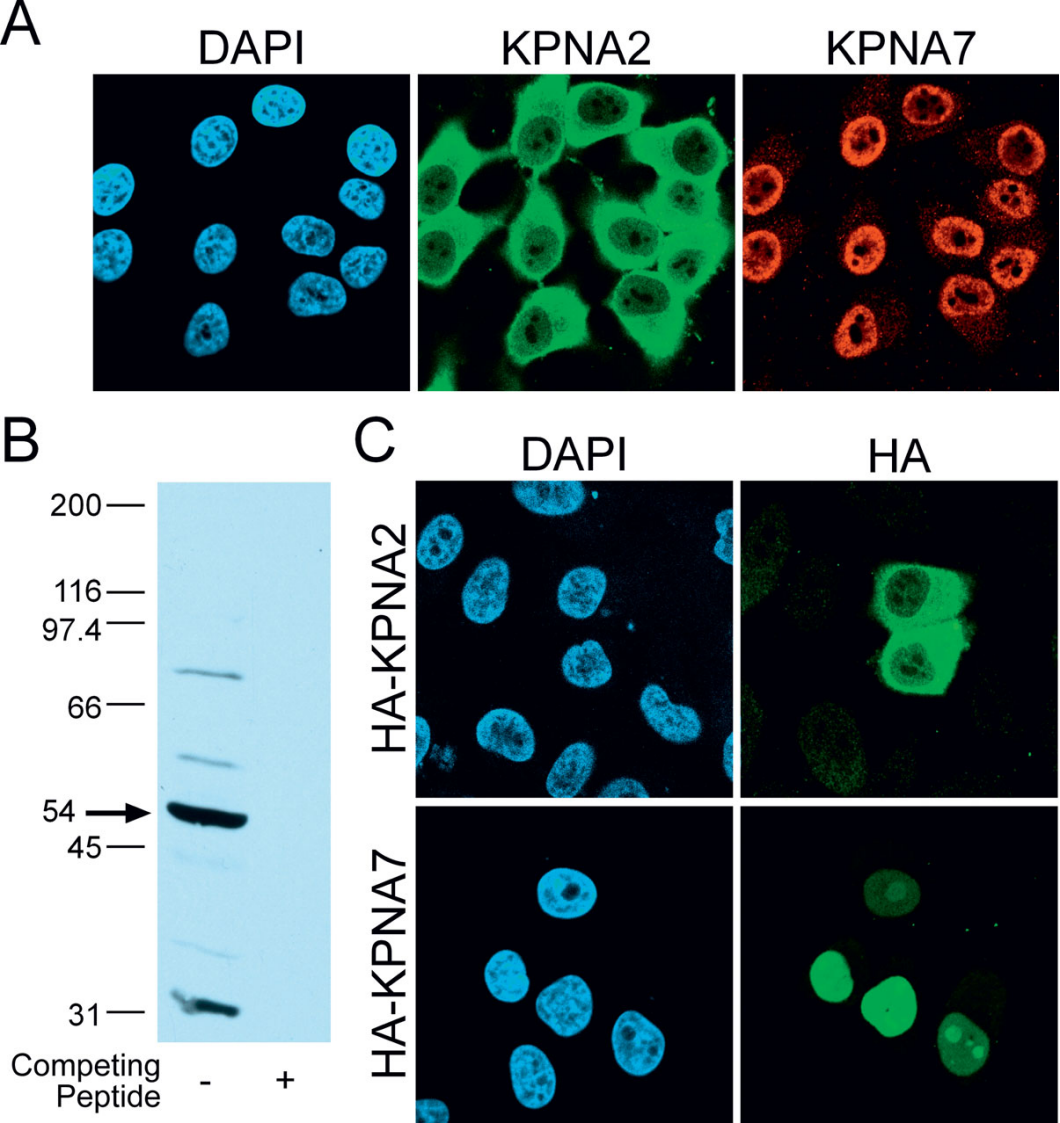
**KPNA7 localizes to the nucleus in HeLa cells**. (A) Immunofluorescence microscopy of endogenous KPNA2 and KPNA7 in HeLa cells shows that KPNA7 is nuclear at steady state. (B) HA-KPNA2 and HA-KPNA7 transfected into HeLa cells, and detected with an anti-HA antibody by immunofluorescence microscopy. (C) Immunoblot of HeLa lysates probed with purified anti-KPNA7, or anti-KPNA7 in the presence of excess KPNA7 peptide to show competition for antibody binding.

The steady state cellular localization of KPNA2 is predominantly cytoplasmic, a distribution that represents the steady state balance between importin α import into the nucleus by importin β, and recycling to the cytoplasm by CAS. In order to examine the steady state localization of KNPA7, we generated an affinity-purified antibody to a unique sequence in its C-terminus (DQDYEFIDYEC). Double-label immunofluorescence microscopy was performed on HeLa cells with the KPNA7 polyclonal antibody and a KPNA2 monoclonal antibody. This revealed that KPNA7 is predominantly nuclear under conditions where KPNA2 is largely cytoplasmic (Fig 6A). By immunoblotting, the KPNA7 antibody detects a 54 kDa protein, which is close to the predicted molecular weight of KPNA7 (57 kDa). Preincubating the KPNA7 antibody eliminates reactivity on immunoblots (Fig 6B Lane 2) and by immunofluorescence microscopy (not shown). To independently assess the localization of these receptors, we transfected HeLa cells with plasmids containing hemagglutinin tagged forms of KPNA2 and KPNA7. Consistent with the results obtained with the endogenous proteins, HA-tagged KPNA2 and KPNA7 localized to the cytosol and nucleus, respectively (Fig 6C).

## Discussion

Here we describe the characterization of a new member of the human importin α family, KPNA7. Sequence analysis revealed that KPNA7 is most similar to KPNA2 (54%), and that it is the most divergent member of the importin α family. Sequence divergence in KPNA7 occurs throughout the protein, including the IBB domain. Our binding data indictates that the IBB of KPNA7 binds importin β stronger than other KPNA proteins. The NLS binding surface is highly conserved in all of the importin α family members, but we observed clear differences among the different family members for binding the RB NLS, SV40 NLS, and the NPM NLS. KPNA7 bound to the RB NLS with equivalent affinity to KNPA2, but failed to bind both the SV40 NLS and the NPM NLS. In addition, by immunofluorescence microscopy KPNA7 localizes to the nucleus, a distribution that contrasts with the predominantly cytoplasmic localization of KPNA2. Our data emphasizes that despite a high degree of structural conservation, there are important differences in the NLS binding specificity and nucleocytoplasmic trafficking of importin -a family members.

Based on the conservation of the residues which make up the RB NLS and SV40 NLS binding surfaces of KPNA2, we reached the conclusion that the NLS binding surface is almost perfectly conserved in all seven members of the importin α family. It was surprising, therefore to observe KPNA-specific binding to both the RB NLS and SV40 NLS. Differences in the IBBs of importin α family members could have potentially altered the affinity for NLS binding through autoinhibition [14], but deletion of the IBB from KPNA7 did not alter its affinity for either the SV40 NLS or the NPM NLS. Assignment of the conserved residues is based upon the ClustalW alignment of the alpha family members, which was then superimposed upon the crystal structure of mouse KPNA2. The key residues are perfectly conserved, but this method of examining the conservation does not take into account gaps, deletions, or the effects of substitutions outside the binding surface on the ultimate structure of the binding surface. There is variability between α family members in the rest of their structure that may result in slight changes in the shape of the binding surface or relative location of the conserved residues which may then alter the NLS specificity of a given importin α. Evidence that the protein context of an NLS plays a role in the specificity for a particular importin α [21] suggests that an otherwise generic NLS may acquire specificity for a specific importin α due to structural constraints imposed by its protein context. Subtle differences in the conformation of the NLS binding surface may therefore lead to specificity for a contextualized NLS, and promiscuous binding to an unstructured NLS encountered out of its proper protein context. These results emphasize the need for experimental validation of predictions made for paralogues. Understanding how highly similar members of the same protein family achieve NLS selective binding will require structural analysis.

KPNA7 is the most divergent of the seven proteins in the human importin α family, and the IBB domain is more divergent than the ARM repeats, which maintain identity in the amino acids necessary for interaction with both the RB NLS and the SV40 NLS. The lack of conservation in the KPNA7 IBB suggests that it does not have the same selective pressures as KPNAs 1 through 6. There are two known functions for the IBB, binding to importin β, and autoinhibition through binding to importin α. Importin β binding could be affected by the amino acid changes in the KPNA7 N-terminus, but our data show very strong binding to importin β. This increased binding to importin β may represent an enhanced affinity of the IBB for importin β, but it could also be due to decreased autoinhibitory binding of the IBB to importin α.

A striking feature of KPNA7 is its localization to the nucleus under steady state conditions, while KPNA2 is predominantly cytoplasmic. This nuclear localization suggests that nucleocytoplasmic shuttling of KPNA7 differs from that of the other members of the importin α family. There are three possibilities for the altered subcellular distribution; 1) enhanced nuclear import by importin β; 2) decreased nuclear export by CAS; and 3) inefficient release of KPNA7 from target NLS, resulting in nuclear retention. The most likely of these situations, given our evidence that KPNA7 binds importin β with much higher affinity than other a family members is that this enhanced binding results in increased nuclear import of KPNA7.

The recent study on bovine KPNA7 showed that it is expressed preferentially in the embryo, suggesting a possible role in early in development [32]. Given that KPNA1 plays a critical role in neuronal differentiation [26], restricted expression of KPNA isoforms could provide a mechanism for selective import of factors that drive key events during development.

## Conclusion

We identified and characterized KPNA7, a highly divergent member of the importin a family of nuclear import receptors. The importin α family in vertebrates comprises seven proteins, KPNA1 through KPNA7. Sequence analysis and molecular modeling revealed the residues that form the NLS binding surface are nearly perfectly conserved throughout the importin a family. Nonetheless, there are striking differences in NLS recognition amongst the different KPNA proteins. Tertiary structure in KPNA proteins could play a major role in creating binding surfaces that can discriminate between different NLS's.

## Methods

### Sequence analysis

RNA from the prostate cancer cell line LnCaP was used as the template to amplify the open reading frame of KPNA7. Multiple alignments were made using the ClustalW alignment tool in Bioedit http://www.mbio.ncsu.edu/BioEdit/BioEdit.html. The Protdist neighbor function was used to create phylogenetic trees, and Dendroscope http://www-ab.informatik.uni-tuebingen.de/software/dendroscope was used to visualize the phylogenetic trees.

### Plasmids

The parent vector used for generating the KPNA proteins was pCMVTNT (Promega). T7 or HA tags were cloned as annealed oligonucleotides with a 5' Kozak sequence into pCMVTNT using Xho1 and EcoR1 sites. KPNAs 1 through 6 were purchased from Origene, PCR amplified and cloned into pCMVTNT-T7 using EcoR1 and Sma1 sites (KPNA1, KPNA2, KPNA3, KPNA5, and KPNA6) or Kpn1 and Sma1 sites (KPNA4). Importin β was cloned using Mlu1 and Xba1 sites.

### Binding Assays

GST fusions of the indicated NLS constructs were expressed and purified by standard methods. His-tagged RanQ69L protein was expressed in the E. coli strain BL21 by induction for 16 hrs at 18 C, and purified on Talon beads. Recombinant Ran protein was loaded with GTP (100 uM) in the presence of EDTA (10 mM) for 20 min at 30 C. Bound nucleotide was stabilized by the addition of excess MgCl2 (20 mM), and unincorporated nucleotide was removed by chromatography on a PD-10 gel filtration column. The pCMVTNT-T7 KPNA constructs were translated *in vitro *with the TNT Coupled Reticulocyte Lysate System (cat# L4610, Promega) using T7 polymerase and ^35^S-methionine (cat# 51006, MP Biomedicals). 10 μg of each GST fusion protein was bound to glutathione beads, and incubated with 250,000 CPM of translated protein for 4 hours at 4°C. The beads were washed with buffer (25 mM Tris pH 7.5, 50 mM NaCl, 5 mM MgCl_2_, 0.1 mM EDTA, 0.5 mg/ml BSA, 0.1% NP-40, 1 mM DTT and protease inhibitors) and eluted in 2x SDS sample buffer. Samples were resolved on 10% acrylamide gels, fixed in methanol/acetic acid, incubated in Autofluor (National Diagnostics), dried and exposed to film at -80°C. Importin β binding to GST-IBB domains was performed in a similar manner except that His-tagged, recombinant importin β (1 μM final concentration) and Ran (15 μM final concentration, loaded with GTP) were used. Binding was assessed by semi-quantitative immunoblotting using IRDye conjugated secondary antibody and the Odyssey infrared imaging system (LI-COR Biosciences, Lincoln, NE).

### Antibody production

A peptide corresponding to a unique sequence (DQDYEFIDYEC) in the C-terminus of KPNA7 was synthesized, coupled to Keyhole Limpet hemocyanin, and injected into rabbits (Cocalico Biologicals, Inc.). Antibody was purified from serum using immobilized peptide.

### Immunofluorescence Microscopy

HeLa cells were grown in DMEM containing 5% Newborn Calf Serum (NCS), 5% Fetal Bovine Serum, 1 mM Sodium Pyruvate at 37°C in 5% CO_2_. For transfection experiments, cells were plated on glass coverslips for 24 hours and transfected with KNPA plasmid using Transfectin (Biorad) according to the manufacturer's protocol. Cells were grown for an additional 24 hours and processed for immunofluorescence microscopy. Coverslips were washed with PBS, fixed with 4% formaldehyde for 20 minutes, permeabilized with 0.5% TritonX-100 for 5 minutes, blocked (2% BSA and 2% NCS in PBS), and incubated with primary antibody. Primary Antibodies used were 16B12 anti-HA antibody (HA.11 16B12, Covance), Rch1 antibody (cat# 610485, BD Biosciences), and anti-KPNA7. Secondary antibodies used were Donkey anti-Mouse FITC (cat# 715-095-150, Jackson ImmunoResearch) and Donkey anti-Rabbit Cy3 (cat# 711-165-152, Jackson ImmunoResearch).

## Authors' contributions

JBK carried out the sequence analysis, molecular modeling, immunofluorescence microscopy, and drafted the manuscript. AMT performed KPNA7 plasmid construction, antibody production and immunoblotting. AS performed the binding assays. DG performed PCR amplification for the KPNA7 transcript. BMP designed the study and edited the manuscript. All authors read and approved the final manuscript.
